# Potential dual inhibitors of PCSK-9 and HMG-R from natural sources in cardiovascular risk management

**DOI:** 10.17179/excli2021-4453

**Published:** 2022-01-05

**Authors:** Mohd Waiz, Sahir Sultan Alvi, M. Salman Khan

**Affiliations:** 1 IIRC-5, Clinical Biochemistry and Natural Product Research Lab, Department of Biosciences, Integral University, Lucknow, U.P. 226026, India

**Keywords:** HMG-R, PCSK-9, statins, PCSK-9 inhibitors, ASCVD, natural secondary metabolites

## Abstract

Atherosclerotic cardiovascular disease (ASCVD) stands amongst the leading causes of mortality worldwide and has attracted the attention of world's leading pharmaceutical companies in order to tackle such mortalities. The low-density lipoprotein-cholesterol (LDL-C) is considered the most prominent biomarker for the assessment of ASCVD risk. Distinct inhibitors of 3-hydroxy-3-methyl-glutaryl-CoA reductase (HMG-R), the chief hepatic cholesterogenic enzyme, are being used since last seven decades to manage hypercholesterolemia. On the other hand, discovery and the association of proprotein convertase subtilisin/kexin type-9 (PCSK-9) with increased ASCVD risk have established PCSK-9 as a novel therapeutic target in cardiovascular medicine. PCSK-9 is well reckoned to facilitate the LDL-receptor (LDL-R) degradation and compromised LDL-C clearance leading to the arterial atherosclerotic plaque formation. The currently available HMG-R inhibitors (statins) and PCSK-9 inhibitors (siRNA, anti-sense oligonucleotides, and monoclonal antibodies) have shown great promises in achieving LDL-C lowering goals, however, their life long prescriptions have raised significant concerns. These deficits associated with the synthetic HMG-R and PCSK-9 inhibitors called for the discovery of alternative therapeutic candidates with potential dual HMG-R and PCSK-9 inhibitory activities from natural origins. Therefore, this report firstly describes the mechanistic insights into the cholesterol homeostasis through HMG-R, PCSK-9, and LDL-R functionality and then compiles the pharmacological effects of natural secondary metabolites with special emphasis on their dual HMG-R and PCSK-9 inhibitory action. In conclusion, various natural products exhibit atheroprotective effects via targeting HMG-R and PCSK-9 activities and lipoprotein metabolism, however, further clinical assessments are still warranted prior their approval for ASCVD risk management in hypercholesterolemic patients.

## Abbreviations

ABCA1 ATP-binding cassette transporter A1

ABCG1 ATP-binding cassette sub-family G member 1

ACAT acyl-CoA: cholesterol acyltransferase

ACC acetyl-CoA carboxylase

ALT alanine Aminotransferase

AMPK AMP-activated protein kinase

ApoB-100 apolipoprotein B-100

ASCVD atherosclerotic cardiovascular diseases

ASOs Anti-sense oligonucleotides

AST aspartate aminotransferase

EGF-A epidermal growth factor like repeat-A

FAS fatty acid synthase

HDL-C high density lipoprotein-cholesterol

HFD high fat diet

HFHC high fat high cholesterol

HMG-R β-hydroxy-β-methyl-glutaryl-CoA reductase

LDL-C low density lipoprotein cholesterol

LDL-R LDL-receptor

LPS lipopolysaccharide

OLZ olanzapine

Ox-LDL oxidized-LDL

PCSK-9 proprotein convertase subtilisin/kexin type-9

PPAR peroxisome proliferators-activated receptor

SCAP SREBP-2 cleavage activating protein

SD Sprague-Dawley

SIRT sertuins

SREBP-2 sterol regulatory element binding protein-2

STZ Streptozotocin

VLDL-C very low-density lipoprotein-cholesterol.

## Introduction

Among other non-communicable disorders (NCDs), atherosclerotic cardiovascular diseases (ASCVD) are the foremost reasons of disease burden and global fatalities (Virani et al., 2021[192]; Ahmad et al., 2020[6]). However, the prevalence of ASCVD cases and associated morbidities is rising day by day in low-income and middle-income countries (LIMCs) including Asian and African continents (Prabhakaran et al., 2018[157]). The severity of the ASCVD is greatly influenced by distinct health behaviors (*i.e*., smoking, nutritional status, alcohol intake and physical inactivity) and other cardiovascular risk factors *i.e*., elevated total cholesterol (TC), triglycerides (TGs), lipoproteins, inflammation, subclinical atherosclerosis, and hypertension (Alvi et al., 2017[16][15]; Virani et al., 2021[192]). Moreover, high level of low-density lipoprotein-cholesterol (LDL-C) has particularly been linked with the above health conditions even sometimes irrespective of the existence of other risk factors (Wang et al., 2019[194]). Circulatory LDL-C are internalized through specialized receptors, namely LDL-receptor (LDL-R). More importantly, the modified variants of LDL, particularly oxidized (Ox-LDL) and glycated adducts (Gly-LDL), have shown the greater ability to initiate the atherosclerotic plaque formation and subsequent ASCVD events, when compared to the unmodified LDL-C (Nabi et al., 2018[142], 2019[143][144]; Wang et al., 2019[194]). 

In contrast, poverty (Jahanihashemi et al., 2018[91]), socioeconomic status (Nguyen and Nguyen, 2020[149]), under-nutrition (Ricci et al., 2019[166]; Nguyen and Nguyen, 2020[149]) and lack of awareness in LMICs also fueled the continuously deteriorating health outcomes irrespective of the efforts made by different government as well as non-government organizations (Tessema et al., 2017[186]). On the other hand, the level of cholesterol is balanced through the co-ordinated regulation of the 3-hydroxy-3-methyl-glutaryl-CoA reductase (HMG-R), the most recognized therapeutic target in the field of cardiovascular medicine (Alvi et al., 2016[18]; Goldstein and Brown, 2015[70]; Nabi et al., 2018[142]). The intracellular HMG-R expression/activity is regulated by an important transcriptional activator *i.e.*, sterol regulatory element binding protein-2 (SREBP-2). The activity of SREBP-2 is triggered by a certain depletion in the circulatory cholesterol level which activates the expression of the key genes liable for the cholesterol synthesis (*i.e.*, HMG-R) as well as receptor-mediated uptake (through LDL-R) in order to amend the diminished intracellular cholesterol levels (Goldstein and Brown, 2015[70]; Ahmad et al., 2020[6]). Increased or uncontrolled activity of HMG-R leads to the build-up of cholesterol in the hepatic cells as well as atherogenic LDL-C in the circulation, thus making it a promising therapeutic target in lipid lowering medications (Goldstein and Brown, 2015[70]). 

Since their discovery, statins are continuously being prescribed for more than seventy years in order to reach the desired cardiovascular outcomes. Despite the desired hypocholesterolemic effects, their life-long prescription at very high doses prompts various undesirable health issues, particularly, rhabdomyolysis, onset of diabetes and associated complications (Careskey et al., 2008; Alvi et al., 2017[16]). Moreover, uneven lipid lowering efficiency and statin intolerance are also the most widely documented deficits of statins. It took almost half of a century after the complex pathway of HMG-R-mediated cholesterol synthesis was elucidated for the scientists to discover another key player in the cholesterol homeostasis. Scientists named this important marker as proprotein convertase subtilisin/kexin type-9 (PCSK-9) (Abifadel et al., 2003[1]; Seidah et al., 2014[172]; Seidah and Prat, 2012[173]). The data from further X-ray crystallography and molecular dynamics studies revealed that PCSK-9 exerts great affinity for epidermal growth factor like repeat-A (EGF-A) of LDL-R (Alvi et al., 2017[15]). Attachment of PCSK-9 to EGF-A restricts the recycling of LDL-R to cell surface as it undergoes lysosomal degradation under the influence of PCSK-9 protease activity resulting in elevated level of LDL-C in the circulation (Lagace, 2014[108]; Lambert et al., 2012[109]; Alvi et al., 2017[16]).

The expression of PCSK-9 is also controlled by the activity of SREBP-2 as well as a specific transcriptional activator hepatocyte nuclear factor-1α (HNF-1α) (Alvi et al., 2017[15]). In contrast, the ability of SREBP-2 to co-stimulate the PCSK-9 and LDL-R mRNA expression limits the therapeutic efficacy of statins which are known to produce their effects via SREBP-2 activation (Welder et al., 2010[201]; Alvi et al., 2017[15]). To overcome these limitations of the statin therapy, researchers are developing PCSK-9 inhibitors using various approaches including siRNA, anti-sense oligonucleotides (ASOs), peptide inhibitors and monoclonal antibodies (mAbs) (Seidah and Prat, 2012[173]). Among distinct approaches, PCSK-9 mAbs (Guedeney et al., 2019[73]) and siRNA inhibitors (Watts and Ockene, 2020[199]; Ray et al., 2018[164]) gained much attention of the world's leading pharmaceutical companies. However, their production and assessment in various clinical trials was withdrawn due to the emerging adverse effects including the formation of anti-drug-antibodies and injection site allergic reactions apart from the expense over the biweekly administration (Wang et al., 2019[193]; van Bruggen et al., 2020[189]).

Considering the adverse effects associated with classical HMG-R and PCSK-9 inhibitors what is needed is the discovery of alternative potential dual inhibitors of these key biomarkers from natural sources. In fact, most of the researchers are turning their backs to the currently available and expensive HMG-R and PCSK-9 inhibitors and shifted their interest towards the alternative medicine in order to suffice the above deficits. In this order, numerous plants including their bioactive compounds have been extensively studied till date for the management of different NCDs in LIMCs, especially, atherosclerosis (Alvi et al., 2015[17], 2016[18]; Wang et al., 2019[194]), diabetes (Akhter et al., 2019[10]; Hashim et al., 2019[81]; Ahmad et al., 2021[7]), cancer (Ahmad et al., 2019[8]), aging (Nabi et al., 2020[145]) and neurodegenerative disorders (Alvi et al., 2019[14]). However, a comprehensive report describing the potential cardioprotective effects of natural secondary metabolites via targeting HMG-R as well as PCSK-9 activity is still lacking. Therefore, the current review firstly describes the mechanistic insights into the cholesterol homeostasis through HMG-R, PCSK-9, and LDL-R functionality and then compiles the cardioprotective effects of natural secondary metabolites with special emphasis on their dual HMG-R and PCSK-9 inhibitory action in ASCVD risk management.

## HMG-R and PCSK-9: Key Regulators of Lipid Metabolism

In mammals, the liver acts as a metabolic power station, where complex cellular processes involved in the cholesterol homeostasis and accumulation in the cells can lead to pathological consequences. Particularly, their entrapment inside the arterial intima may cause ASCVD through a series of oxidative modifications, inflammatory cascades, and immunological responses (Trapani et al., 2012[188]; Ahmad et al., 2020[6]; Wang et al., 2019[194]). A number of successive findings led to the understanding of current scenario of cholesterol metabolism (Goldstein and Brown, 2015[70]). It is now clear that the level of cholesterol is maintained by interplay between de novo biosynthesis, uptake, export, and storage. The HMG-R, chief enzyme of lipid homoeostasis, coverts the HMG-CoA into mevalonic acid with the use of 2 molecules of NADPH as a reducing agent (Luo et al., 2020[132]). Generally, the cells maintain cholesterol level either by HMG-R-mediated endogenous cholesterol synthesis or receptor-mediated uptake. The expression of HMG-R is tightly regulated via SREBP-2. Cholesterol depletion to a certain threshold causes SREBP cleavage activating protein (SCAP) to interact with the COPII which is responsible for the transportation of protein from endoplasmic reticulum (ER) to Golgi apparatus (Goldstein and Brown, 2015[70]).

**Figure 1:**
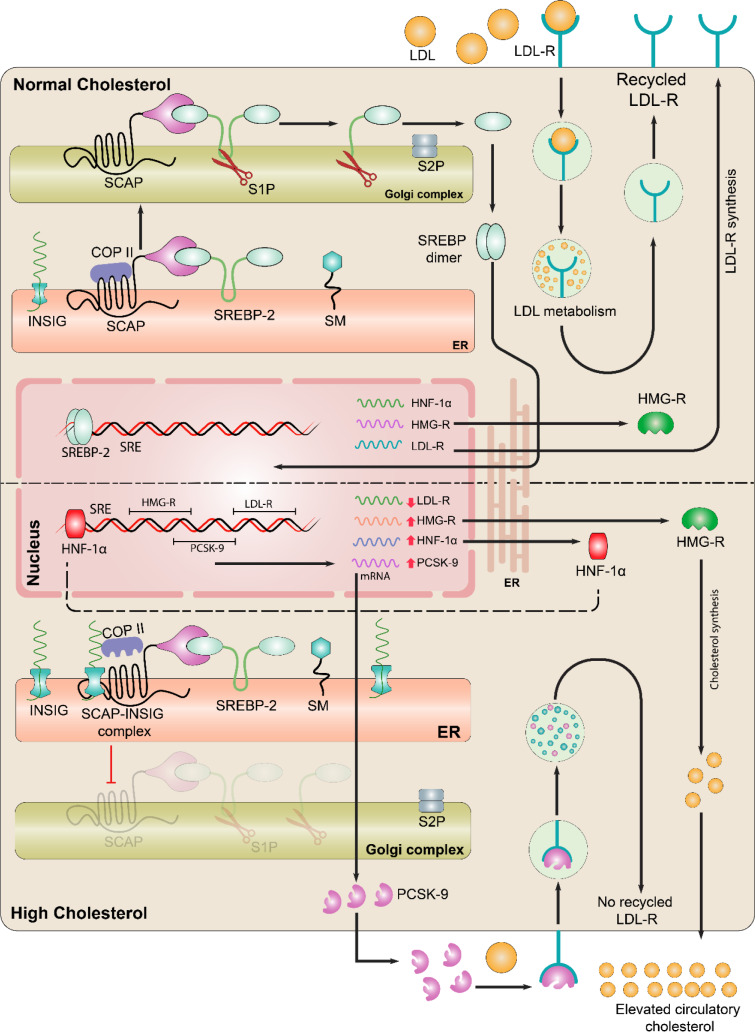
Mechanistic insight into the cholesterol homeostasis: Sterol regulatory element binding protein (SREBP-2) is the key modulator of cholesterol biosynthesis. Endoplasmic reticulum (ER) is in center where SREBP-2 is synthesized but for the activation, its translocation is required to the Golgi. In the ER, SREBP-cleavage activating protein (SCAP) interacts with the SREBP-2. Upper panel: In case of normal cholesterol in ER, SCAP interacts with SREBP-2, allowing COPII to bind SCAP which is responsible for the movement of SCAP-SREBP complex from ER to Golgi. In the Golgi SREBP-2 undergoes proteolytic cleavage by site 1 protease (S1P) and S2P, thereby releasing the active domain that enters into nucleus and binds with the sterol regulatory element (SRE) in the promoter, eventually, activates transcription of target genes such as HMG-R, LDL-R, and PCSK-9. HMG-R normally regulates the cholesterol biosynthesis. LDL-Rs are present on the surface of hepatocytes that clear the excess cholesterol via adopting LDL particle and leads to the lysosomal degradation. After the degradation of LDL, the LDL-R are recycled back to the surface of hepatocyte for repeated clearance of circulatory LDL. Lower panel: In case of excess cholesterol, INSIG binds to the SCAP and detachment of COPII occurs. Due to the detachment of COPII, SREBP-2 are retained in ER which restricts further processing of SREBP-2. Lack of processed SREBP-2 under elevated cholesterol conditions results in altered HMG-R and LDL-R expression as well as activity, whereas, HNF-1α continues to stimulate the expression of PCSK-9, which binds to the LDL-R and leads to its lysosomal degradation that restricts the LDL clearance from the blood.

SREBPs are cleaved in the Golgi apparatus and resulted N-terminal of SREBP migrates to the nucleus, where it regulates the expression of genes essential for cholesterol synthesis (*i.e.*, HMG-R) and receptor-mediated uptake (*i.e.*, LDL-R) (Ahmad et al., 2020[6]; Goldstein and Brown, 2015[70]; Alvi et al., 2017[15]). Instead, in case of excess cholesterol in ER, cholesterol binds to the SCAP causing its attachment with INSIG, which further leads to the detachment of COPII protein from the SREBP/SCAP-complex and limits its transport to the Golgi. The decline in processed nuclear SREBP alters the transcription of genes involved in the intracellular cholesterol biosynthesis and uptake (Goldstein and Brown, 2015[70]). LDL-R is a major player responsible for the uptake of cholesterol, namely LDLs, via peripheral cells from the circulation. LDL-Rs exist at the surface of the plasma membrane of most cells but the most preferential expression has been reported in case of hepatic cells. The circulatory LDL is captured by the LDL-R, internalized and proceeds to the lysosomal degradation, however, LDL-R is recycled back to the cells surface for repeated LDL clearance (Luo et al., 2020[132]). The major mechanistic insights of cholesterol homeostasis have been shown in Figure 1(Fig. 1).

Despite the mystery of cholesterol homeostasis and suddenly encountered heart attacks was resolved by the pioneering workers in the field, the uneven lipid lowering with statins and statin-intolerance raised potential concerns regarding the involvement of other regulatory factors which possibly had detrimental part in lipid homeostasis in certain individuals. It was most frequently observed that some patients were poorly responding to the statin treatment as reflected by their elevated LDL-C level, even used at maximal doses. These uncertain effects of statins were explained with the discovery of PCSK-9, one of the 9 members of proprotein convertases (serine proteases) (Alvi et al., 2017[16]). In addition to a catalytic and a C-terminal domain, it contains an inactive pro-domain which is catalytically cleaved in order to get matured and functionally active PCSK-9. The data from X-ray crystallography and molecular dynamics studies revealed that it has great affinity for EGF-A domain of LDL-R (Alvi et al., 2017[16]). 

Attachment of PCSK-9 to EGF-A restricts the recycling of LDL-R to cell surface as it undergoes lysosomal degradation under the influence of PCSK-9 protease activity resulting in elevated level of LDL-C in the circulation (Lagace, 2014[108]). The extent of the PCSK-9 expression relies on the activity of its transcriptional activators *i.e*., SREBP-2 and HNF-1α, where the former co-stimulates the expression of both LDL-R and PCSK-9, whereas the latter is reckoned to strongly activate the expression of PCSK-9 making it a prime target in lipid lowering goals (Li et al., 2009[119]; Alvi et al., 2017[15]; Ahmad et al., 2020[6]). 

## Targeting PCSK-9 and HMG-R: Classical Inhibitors and Mechanisms

### HMG-R inhibitors

Statins are the class of drugs which were first ever approved as HMG-R inhibitors by US Food and Drug Administration (FDA) due to their competitive inhibitory effects against HMG-R activity as well as desired lipid lowering in patients with poor lipid metabolism (Nabi et al., 2018[142]). The binding efficiency of statins with the HMG-R active pocket was reported to be almost 10 thousand times greater than that observed in the case of the original HMG-R substrate, *i.e*., HMG-CoA, resulting in its restricted binding to the enzyme as well as intracellular cholesterol biosynthesis (Istvan and Deisenhofer, 2001[90]). These classical HMG-R inhibitors showed promising lipid-lowering effects in hypercholesterolemic patients as intra-cellular hepatic cholesterol constitutes the majority of the atherogenic load in the circulation (Stancu and Sima, 2001[182]). Till date, various statins have been discovered and approved for clinical implications in order to manage ASCVD worldwide and to reduce the ASCVD-associated mortality, however, their distinct structures contribute to varying binding patterns against HMG-R active pocket, pharmacokinetics, bioavailability as well as lipid-lowering efficiencies (Graham et al., 2007[71]; Nabi et al., 2019[143]). 

### PCSK-9 inhibitors

The direct association of PCSK-9 and elevated level of LDL-C with high ASCVD mortality was established since its discovery in the very beginning of the 21^st^ century (Wang et al., 2019[194]; Alvi et al., 2017[16]). Studies in a quick succession revealed that PCSK-9 interacts with EGF-A of LDL-R to mediate its intracellular degradation, hence limits the recycling of these receptors responsible for atherogenic lipoprotein clearance from the blood-stream (Leren, 2014[117]; Horton et al., 2007[84]). Considering the regulatory role in LDL-R-mediated lipoprotein clearance from the peripheral blood, PCSK-9 has been established as one of the key molecular targets in the field of cardiovascular pharmacology (Latimer et al., 2016[115]; Farnier, 2016[64]; Ahmad et al., 2020[6]). In this context, distinct approaches have been implied to restrict the PCSK-9 expression (mRNA) and functionality (protease activity) including small interfering RNA (siRNA) (Frank-Kamenetsky et al., 2008[66]), antisense oligonucleotides (ASOs) (Graham et al., 2007[71]), inhibitory peptides (Lammi et al., 2016[111]), EGF-A mimetics, monoclonal antibodies (mAbs) (Guedeney et al., 2021[74], 2019[73]) and small molecule inhibitors (both synthetic and natural) (Alvi et al., 2017[16][15]; Wang et al., 2019[194]). Among these, siRNA and ASOs have been proven to efficiently restrict the PCSK-9 expression at transcriptional level (Frank-Kamenetsky et al., 2008[66]; Graham et al., 2007[71]), whereas, mAbs are known to limit the PCSK-9 functionality post-translationally (Guedeney et al., 2021[74]). 

The siRNA approach has shown promising diminution in the level of PCSK-9 and LDL-C via suppression of PCSK-9 expression in a primate model which ultimately enhanced the availability of recycled LDL-R (Frank-Kamenetsky et al., 2008[66]). Restricting the PCSK-9-EGF-A complex using mAbs was also extensively studied and outcomes were suggestive of a marked decline in circulatory LDL-C level (~80 %) (Duff et al., 2009[58]; Chan et al., 2009[40]). 

## Limitations of Classical PCSK-9 and HMG-R Inhibitors

Prescription of statins is known to lower the cholesterol through HMG-R inhibition as well as the up-regulation of the LDL-R expression which ultimately directs increased clearance of LDL-C from the bloodstream (Seidah and Prat, 2012[173]). Statins are one of the highest sold drugs in the world due to their long-lasting prescription for the patients suffering either with heart attacks and hypercholesterolemia, however, their life-long oral administration leads to various undesirable health issues, particularly, rhabdomyolysis and onset of diabetes and associated complications (Careskey et al., 2008[38]; Alvi et al., 2017[16]). Moreover, uneven lipid lowering efficiency and statin intolerance are also the most widely documented deficits of statins. In contrast, co-stimulation of PCSK-9 and LDL-R via a common transcriptional activator, *i.e.*, SREBP-2, in statin treated patients limited the efficacy of these classical HMG-R inhibitors (Careskey et al., 2008[38]; Seidah and Prat, 2012[173]). On the other hand, inhibitors of PCSK-9 have been used to effectively lower the cholesterol content as a replacement for classical HMG-R inhibitors, particularly in the patients facing statin intolerance and inadequate cholesterol regulation (Weisshaar and Zeitlinger, 2018[200]). Although mABs, siRNA, and ASOs have shown great potential in terms of lipid lowering, their long-lasting prescription has raised significant concerns including expensiveness, repeated administrations, and injection site irritations. Long duration clinical trials have also documented that the administration of these mABs, siRNA, and ASOs in most of the individuals elicit the formation of anti-drug antibodies to a significant titre (Gupta et al., 2010[77]; Ray et al., 2017[163]; van Bruggen et al., 2020[189]). 

## Natural Products: Novel Alternative Potential Dual Inhibitors of PCSK-9 and HMG-R

**Figure 2:**
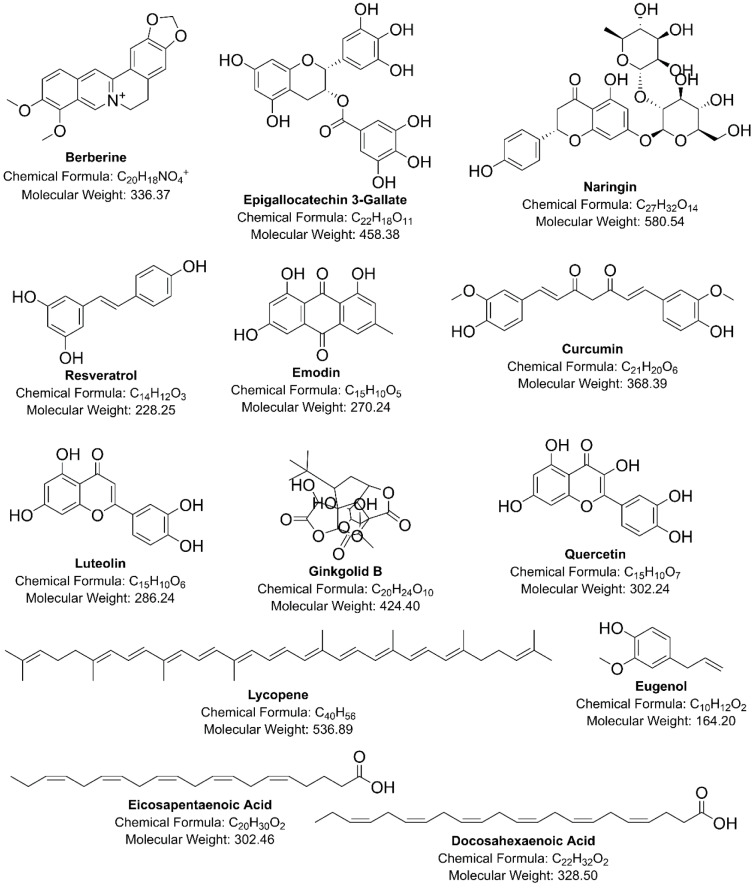
Chemical structures of natural inhibitors of PCSK-9 and HMG-R

To overcome the above-discussed deficits of classical PCSK-9 and HMG-R inhibitors on health and to meet the desired health goals and public priorities in terms of safety and cost-related issues, researchers in the last decade focussed their attention on novel natural therapeutic candidates possessing dual inhibitory activity against PCSK-9 and HMG-R at transcriptional as well as post-translational stages (Alvi et al., 2017[16][15]; Lammi et al., 2016[111]; Wang et al., 2019[194]). The chemical structures of natural compounds that act as dual-inhibitors of PCSK-9 and HMG-R have been shown in Figure 2(Fig. 2). Some of the dual inhibitors of PCSK-9 and HMG-R from natural origins and their lipid-lowering mechanisms are appended below: 

### Berberine

The berberine (BBR), a bioactive metabolite isolated from *Berberis Spp.* particularly *B. aristata* and *B. vulgaris*, is well reckoned for its beneficial pharmacological effects (Potdar et al., 2012[156]). An *in vitro* study showed that the treatment with BBR markedly curbed the expression of PCSK-9 mRNA in HepG2 cells, which ultimately restricted the PCSK-9 protein secretion from the HepG2 cells by 87 % (Cameron et al., 2008[35]). In the same study, the researchers also revealed that the level of LDL-R mRNA expression was up-regulated dose-dependently in HepG2 cells (Kong et al., 2004[106]; Cameron et al., 2008[35]). The BBR also increased peroxisome proliferator-activated receptors-α (PPARα) mRNA and SREBP-2 mRNA expression by 39 % and 74 % respectively. In the same study, researchers also reported that BBR was not involved directly into the alteration of stability of PCSK-9 mRNA while reducing its promoter activity through HNF-1α (Cameron et al., 2008[35]). The extracellular signal-regulated kinase (ERK)-dependent PCSK-9-lowering effect of BBR metabolites was also observed, where berberrubine and its analogues were most potent (Cao et al., 2019[36]).

The first *in vivo *substantiation of hypocholesterolemic activity of BBR was reported in a study conducted on high fat high cholesterol (HFHC) fed female golden hamster rats (Kong et al., 2004[106]). Briefly, Serum TC as well as LDL-C was reduced while the expression of LDL-R mRNA was elevated in a dose- and time-dependent fashion after 10 days treatment of BBR. BBR treatment also reduced the LDL-C level by 26 % (at 50 mg/kg/day) and 42 % (at 100 mg/kg/day), as compared to the untreated rats (Kong et al., 2004[106]). Though, the initial *in vivo* report regarding the outcome of BBR on PCSK-9 was assessed in lipopolysaccharide (LPS)-induced inflamed liver of dyslipidemic C57BL/6 mice model (Xiao et al., 2012[211]). This study concluded that oral administration of BBR significantly lowered the PCSK-9 mRNA expression in a dose-dependent fashion, whereas, an up-regulation in LDL-R mRNA expression was also observed (Xiao et al., 2012[211]). In contrast, another study in HFD-induced obese Sprague-Dawley (SD) rats demonstrated that BBR markedly suppressed the expression of PCSK-9 through HNF-1α, whereas, the expression of LDL-R mRNA was up-regulated through the activation of its transcriptional activator *i.e*., SREBP-2. Such regulation of PCSK-9 and LDL-R expression via BBR treatment resulted in amelioration in the level of serum TG, TC, LDL-C and HDL-C level in these obese rats (Jia et al., 2014[96]; Dong et al., 2015[55]). Similar findings regarding the effect of BBR on lipid and lipoprotein level as well as hepatic PCSK-9 mRNA expression were reported in another study in HFD-induced hypercholesterolemic rats (Liu et al., 2015[128]).

Another study conducted in HFD administered SD rats demonstrated that BBR (200 mg/kg/day) lowered the hepatic cholesterol content and modulated the expression of LDL-R, HMG-R as well as apolipoprotein E (ApoE) mRNA (Chang et al., 2012[42]). A further *in vivo* study validated that intraperitoneally administered BBR (5 mg/kg/day) reduced the HMG-R activity in the liver of SD rats (Wu et al., 2011[209]). Recently, an *in vivo* study confirmed the lipid lowering effect of BBR via reduction in TC, apolipoprotein-B 100 (ApoB-100), and VLDL-C in HFD-induced mice but for the reduction in LDL-C high dose of BBR was required. The treatment with BBR also significantly reduced the pro-inflammatory cytokines like tumor necrosis factor alpha (TNF-α), interleukins IL-1β, IL-6 and the slight hike in level of adiponectin was observed in ApoE-/- C57BL/6J mice (Wu et al., 2020[208]). BBR suppressed the HMG-R mRNA expression dose-dependently in HepG2 cells, however, the expression of two other cholesterogenic enzymes, namely, farnesyl-diphosphate synthase and 7-dehydrocholesterol reductase mRNA was unaffected (Cameron et al., 2008[35]). 

The cells treated with BBR exhibited decreased intracellular TGs content and intracellular lipid level via the regulation of AMPK pathway (Cao et al., 2013[37]). Another *in vitro* study was performed to uncover the lipid lowering mechanism of BBR on olanzapine (OLZ)-induced adipogenesis in 3T3-L1 cell model. In this attempt, berberine reduced expression of SREBP-1, fatty acid synthase (FAS), PPAR-γ, SREBP-2, LDL-R, and HMG-R in OLZ-induced adipogenesis 3T3-L1 cells. Besides animal studies in hamsters (Brusq et al., 2006[32]), rats (Jia et al., 2008[95]; Jin et al., 2010[98]) and mice (Chueh and Lin, 2011[46]), the hypolipidemic effectiveness of BBR was also investigated in the individuals with hypercholesterolemia facing statin intolerance and reported that BBR administration reduced the TG and LDL-C level by approximately 13-30 % and 20-25 %, respectively (Barrios et al., 2017[26]). Another clinical study was performed on 97 mild hyperlipidemic patients at a dose of 300 mg BBR or placebo for 3 months. After the treatment with BBR, the TC, TG, and LDL-C level was reduced and the HDL-C level increased. Berberine was also effective in improving lipid level in mildly hyperlipidemic patients (Wang et al., 2016[196]). 

In another study, BBR containing nutraceutical pill (500 mg) or ezetimibe (10 mg) were tested as alternative treatments for 6 months in 228 primary hypercholesterolemic patients with statins intolerance. BBR was shown to be more effective than ezetimibe in lowering LDL-C and improving statin intolerance (Pisciotta et al., 2012[154]). The major meta-analysis (27 clinical studies with 2,569 participants) concluded that BBR lowered the TG, TC, and LDL-C levels while increased the HDL-C levels (Lan et al., 2015[114]). A recent enzoinformatics study showed that BBR exerted strong binding interaction via a significant number of hydrogen bonds with the amino acid residues Asn755, Glu559 and Lys735 Ser565 of the HMG-R active pocket (Ghareeb et al., 2015[68]).

### Lupin

Lupin, a protein-rich grain legume from the Fabaceae family, is extensively expressed by four domestic cultivars *i.e., L. luteus* (yellow lupin), *Lupinus albus* (white lupin), *L. mutabilis* (pearl lupin) and *L. angustifolius* (sweet leaf lupin; Fabaceae). Proteins present in lupin have been studied for years, notably for their ability to lower the levels of plasma cholesterol, which is due to its LDL-R stimulating mechanism (Sirtori et al., 2004[180]). Lupin from the *L. albus* reduced cholesterolemia in atherosclerosis-induced rabbit model and was found to be protective against atherosclerosis progression (Marchesi et al., 2008[136]). After four-week of supplementation, lupin significantly lowered the plasma cholesterol in adolescents. On the other hand, in the same study, the researchers for the first time reported that consuming food enriched with lupin reduces TC, LDL-C, TGs, homocysteine, and uric acid (Bähr et al., 2013[25], 2015[24]; Lammi et al., 2016[111]). Lupin also reduced the PCSK-9 protein level via down-regulation of HNF-1α in hepG2 cells (Lammi et al., 2016[113]). A randomized trial showed that dietary intake of lupin by metabolic syndrome patients reduced the LDL-C and PCSK-9 levels (Pavanello et al., 2017[152]). A recent study uncovered that lupin maintains the cholesterol homeostasis in HepG2 cells via suppression of PCSK-9 and HNF-1α levels (Lammi et al., 2019[110]). 

Furthermore, GQEQSHQDEGVIVR (T9), a peptide derived from the lupin, was found to be the best amongst other lupin peptides which enhanced the LDL-C uptake in a dose-dependent manner via inhibiting the interaction between PCSK-9 and LDL-R and also reduced the PCSK-9 protein expression. Thus, inhibition of interaction between PCSK-9 and LDL-R and reduction in the HNF-1α expression were two main activities of lupin protein PCSK-9 in lipid homeostasis. In the same study, researchers also explored the *in* *silico* interaction of lupin peptide and PCSK-9 and found that this peptide efficiently interacted with PCSK-9 through Glu3, His6, Gln7, Asp8, Ile12, Cys378, Glu195, Asp238, Ser153, and Lys243 (Lammi et al., 2016[111]). On the other hand, the same T9 peptide was further evaluated for its HMG-R inhibitory activity. The findings from this study revealed that the exposure to T9 peptide inhibited the action of HMG-R in HepG2 cells that were transfected with the mutant PCSK-9 with an IC_50 _value of 99.5 µM. Further, T9 peptide boosted the LDL-R level by inhibiting the HMG-R and improved extracellular LDL-uptake in HepG2 cells and exhibited potent hypocholesterolemic effects (Lammi et al., 2019[110]). Another peptide YDFYPSSTKDQQS obtained through the pepsin-mediated hydrolysis of lupin also inhibited the HMG-R. This peptide from lupin also increased the LDL-R level by SREBP activation, as a result, LDL-uptake was increased in HepG2 cells (Lammi et al., 2018[112]).

### Quercetin

Quercetin (QRC), a natural flavonoid present in fruits and vegetables, has distinct bioactivities comprising anti-carcinogenic, anti-inflammatory, antiviral, antioxidant, neuroprotective, as well as the ability to slow down lipid peroxidation (Aguirre et al., 2011[5]; El-Saber Batiha et al., 2020[61]). The naturally occurring dominant form of QRC glycosides is deglycosylated in the intestine, resulting in a QRC-free form that is a substrate for liver enzymes which is accountable for the generation of distinct QRC metabolites (Santangelo et al., 2019[170]; Dabeek and Marra, 2019[52]). A few formulations of QRC have been made to improve the bioavailability and prevent its degradation (Zhao et al., 2019[220]; Riva et al., 2019[167]). QRC glycosides are the most abundant flavonol molecules found in plants such as berries, apples, capers, grapes, tomatoes, onions,* Brassica* vegetables, shallots, tea, and nuts. Quercetin is also present in medicinal therapeutic herbs, such as *Ginkgo biloba*, *Hypericum perforatum*, and *Sambucus canadensis *(Häkkinen et al., 1999[79]; Williamson and Manach, 2005[204]; Wiczkowski et al., 2008[203]). As a key hypolipidemic mechanism, the LDL-R gene expression was found to be increased via QRC treatment in hepatic cells, resulting in clearance of circulating LDL. This influence is evidently regulated by activating the SREBP-2 (Moon et al., 2012[140]).

An *in* *vitro* study reported that the treatment with the glycosylated form of QRC reduced the level of PCSK-9 mRNA by 20-30 % in HepG2 cells in a dose-dependent fashion (Mbikay et al., 2014[139]). Interestingly, 20 µM of QRC also affected PCSK-9 expression in foam cell macrophages model ( Li et al., 2018[123]; Adorni et al., 2017[2], 2020[3]). Conversely, quercetin-3-glucoside (Q3G) induced the PCSK-9 and LDL-R expression in pancreatic cells of mouse. However, this has been regarded as a beneficial outcome. In fact, quercetin may limit the cholesterol uptake by increasing PCSK-9 level relative to LDL-R, preventing cholesterol-dependent dysfunction in these cells (Mbikay et al., 2018[138], 2014[139]).

As the level of circulating cholesterol partially depends on the PCSK-9 and LDL-R produced by liver, it should be emphasized that the preceding *in vitro *studies strongly limited by increased concentration of QRC and the usage of its glycosidated form rather than the free aglycone. Another study concluded that the supplementation of QRC significantly diminished the augmented level of TC, LDL-C, TNFα, IL-10, IL-6 and PCSK-9 in HFD-induced atherosclerotic ApoE-/- mice (Li et al., 2020[124]). Further* in vivo* investigation observed that QRC treatment for three months down-regulated the PCSK-9 expression and up-regulated the LDL-R expression in the hepatocytes and aorta of HFD-induced mice (Jia et al., 2019[94]; Mbikay et al., 2018[138]).

On the other hand, a study reported that QRC treatment reduced the HMG-R activity resulting in reduction in overall cholesterol synthesis in C6 glioma cells of rats. QRC treatment also caused 39 % reduction in HMG-R mRNA and 20 % in acetyl-CoA carboxylase-1 mRNA expression but the expression of fatty acid synthase mRNA was not affected (Damiano et al., 2019[53]). A recent study also delineated the role of QRC on lipid metabolism in LDL-induced HepG2 cells and reported that QRC at varying concentrations (1-90 μM) had no inhibitory effect on cell viability. The reduction in droplets of lipids and TC was observed in a dose- and time-dependent fashion in QRC treated HepG2. In the same study, the depletion in SREBP-2 and HMG-R was also observed (Hu et al., 2020[85]). One more cell-based study reported that QRC lowers the cholesterol via direct inhibition of HMG-R in HepG2 cells (Cuccioloni et al., 2016[48]).

Apart from the *in vitro* cell culture studies, an* in vivo* report also demonstrated that QRC diminishes the plasma cholesterol, TG, LDL-C, and thiobarbituric acid reactive substances (TBARS) in tyloxapol-induced hypercholesterolemic male Wistar rats. Additionally, reduction in HMG-R activity was also observed, while the LDL-R expression was up-regulated (Khamis et al., 2017[103]). A recent study further delineated the regulatory role of QRC in cholesterol homeostasis in diabetic mice model in which QRC modulated the expression of key mediators like LDL-R, HMG-R, SREBP-2 and SCAP along with the improvement in lipids/lipoproteins content (Jiang et al., 2019[94]). The molecular simulation studies also validated the strong HMG-R inhibitory activity of QRC as it strongly occupied the catalytic site of HMG-R via masking its Asn658, Gly656, Val805, Gly808, and Gly560 residues with significant number of hydrogen bonds (Islam et al., 2015[89]; Cuccioloni et al., 2016[48]).

### Epigallocatechin-3-gallate (EGCG)

Epigallocatechin-3-gallate (EGCG) is a naturally occurring vital catechin commonly extracted from green tea [*Camellia sinensis* L. Ktze. (Theaceae)]. It has many biological properties including antioxidant, cancer chemoprevention, anti-atherosclerotic effect, radiation protective and also helpful in burning fat (Nagle et al., 2006[146]). In a cell-based study, EGCG showed anti-hypercholesterolemic activity in HepG2 cells with the elevated expression of LDL-R mRNA via activating the ERK signaling pathway. Moreover, the ubiquitin lysosomal degradation of ApoB was also enhanced by EGCG (Li et al., 2006[120]). This action was found to be independent of the 67 kDa laminin receptor, a well-studied EGCG receptor. The researchers also demonstrated that EGCG down-regulates the cholesterol production via attenuation of SREBP-2 with the activation of sirtuin-1/forkhead box protein O1 (SIRT1/FOXO1) signaling pathway (Zanka et al., 2020[218]; Li and Wu, 2018[125]).

EGCG also up-regulated the LDL-R expression at both mRNA and protein level, whereas, the extracellular level of PCSK-9 protein was significantly reduced but the mRNA level of PCSK-9 was unchanged in HepG2 cells. In addition, statin-derived stimulation of PCSK-9 level was also attenuated by EGCG (Kitamura et al., 2017[105]). Moreover, EGCG supplementation decreased the circulating PCSK-9 level as well as stimulated the expression of LDL-R in HFD-induced rats via blocking the HNF-1α and activating FOXO-3a. Similar findings were reported concerning the outcome of EGCG on the expression level of PCSK-9 in HepG2 cells (Cui et al., 2020[51]).

In addition to the effects of EGCG on PCSK-9-LDL-R pathway, an *in vivo* study concluded that the supplementation of EGCG in 1,3-dichloro-2-propanol (1,3-DCP)-induced C57BL/6J mice significantly reduced the hepatic lipid accumulation and also modulated the level of lipids/lipoproteins and the activities of alanine aminotransferase (ALT) and aspartate aminotransferase (AST). Moreover, the mRNA expression of SREBP-2 and HMG-R was reduced via EGCG (Lu et al., 2018[131]). Additionally, EGCG strongly inhibits the HMG-R activity. The same study also opted the molecular docking simulations to reveal the binding pattern of EGCG with crystal structure of human HMG-R. In this attempt, the EGCG was found to strongly bind to the HMG-R via stable hydrogen bonding with Glu559, Asp690, Lys691, Gln770, Val805, Gly807, and Met659, Met655, Asn658, and Asp767 residues (∆G: −11.30 kcal/mol) (Cuccioloni et al., 2011[49]). Another *in vitro* study assessed the effect of coadministration of EGCG and pravastatin on HMG-R activity. The combined effects of EGCG and pravastatin suppressed HMG-R more effectively than the effects of each individual inhibitor (Cuccioloni et al., 2011[49]).

Similarly, one more cell culture-based study showed that the inhibition of HMG-R activity via EGCG resulted in the decreased cholesterol content in HepG2 cells, whereas, the HMG-R mRNA was unaffected after EGCG treatment. These findings were further validated through molecular docking studies which revealed that EGCG binds efficiently inside the active pocket of HMG-R (∆G: -41.87 kcal/mol) (Wu et al., 2014[210]).

### Resveratrol

Resveratrol (3,4′,5-trihydroxystilbene) (RSV) is a natural phytoalexin produced by some phanerogams such as grapevines and also found in red wine, grapes, and peanuts (Frémont, 2000[67]). RSV, being lipophilic in nature, accumulates in distinct tissues and organs including brain, liver, and intestine. In plasma, urine and several tissues of humans, about 20 RSV-derived metabolites have been identified, whereas, resveratrol-3-O-sulfate was found to be the most abundant hepatic as well as circulating metabolite (Singh et al., 2019[179]). Treatment with trans-RSV induced hepatic LDL-R expression by proteolytic activation of SREBPs in HepG2 cells and reduced the expression of ApoB-100 which was independent from the adenosine monophosphate-activated protein (AMP) kinase (AMPK)-mediated signaling pathway (Yashiro et al., 2012[216]). RSV too reduced the SREBP-1c at both mRNA and protein levels. An *in* *vitro *report depicted that PCSK-9 expression was reduced via the down-regulation of SREBP-1c expression and up-regulation of LDL-R in HepG2 cells (Jing et al., 2019[99]).

On the other hand, polydatin (PD), a glucoside of RSV, was recognized as the inhibitor for the PCSK-9/LDL-R interaction. In insulin resistant HepG2 cells, PD enhanced the level of LDL-R by preventing its lysosomal degradation associated with PCSK-9 (Wang et al., 2016[198]). The *in silico* study showed that PD effectively binds to the active pocket site of PCSK-9 through an appropriate count of hydrogen bonds with Cys358, Val 435, Asn439, and Asp651 residues. This data suggested that binding of PD with PCSK-9 restricted the interaction between PCSK-9 and LDL-R resulting in enhanced clearance of circulating LDL. Moreover, the treatment with PD (100 mg/kg) for 4 weeks markedly inhibited PCSK-9 expression and also increased protein levels of LDL-R in C57BL/6 mice (Wang et al., 2016[197]). 

A study showed that RSV administration reduced the concentration of plasma TG, TC, and LDL-C in hepatic tissue of HFD-fed male Syrian Golden hamsters. ApoB content was also reduced in RSV administered rats. Another animal based study suggested that intake of RSV reduces the serum cholesterol through down-regulating the hepatic HMG-R mRNA expression in rats (Cho et al., 2008[43]). Moreover, RSV supplementation also enhanced the hypocholesterolemic action of pravastatin through the inhibition of HMG-R. A recent study in obese HFD-induced C57BL/6J hyperlipidemic mice reported that RSV enhanced the expression of PPARα, LDL-R, and significantly down-regulated the HMG-R mRNA expression (Guo et al., 2017[75]). In a cell-based study, RSV reduced the HMG-R mRNA expression as well as its activity by 30 % in the theca-interstitial cells of SD female rats (Wong et al., 2011[205]). Another *in vitro* study in human endometrial biopsies reported that RSV reduced the synthesis of cholesterol and also reduced the HMG-R mRNA expression level by 30 % (Villanueva et al., 2013[191]).

On the other hand, a preclinical study showed that RSV restores the normal level of lipid by modulating the level of TG, LDL-C, and HDL-C (Göçmen et al., 2011[69]). Clinically, a meta-analysis showed that RSV supplementation for 3 weeks markedly diminished the fasting glucose, TC, and LDL-C levels (Guo et al., 2018[76]). An *in vivo *study in HFD-induced hypercholesterolemic animals (both rats and rabbits) showed that administration of PD reduced the level of serum TC, TG, LDL-C as well as ApoA-1, whereas HDL-C level was increased (Xing et al., 2009[213]; Du et al., 2013[57]). According to a study, PD controls the activity of the enzymes responsible for hepatic cholesterol homoeostasis via modulating the SREBP-2 expression (Zhang et al., 2012[219]; Alvi et al., 2017[16]). In the same context, Browning and Horton demonstrated that PD significantly reduced the HMG-R activity and the level of serum TC, TG, and LDL-C and also enhanced the expression of LDL-R in hepatocytes (Browning and Horton, 2004[31]). An *in vitro* study explored the HMG-R inhibitory potential of *Melinjo* (*Gnetum gnemon L*.) seed extracts (n-hexane, dichloromethane (DCM), ethyl acetate, methanolic, aqueous), in which DCM and ethyl acetate extracts showed maximum HMG-R inhibitory activity.

Further, UPLC/MS analysis of DCM extract was performed to identify the bioactive compounds in it. In this attempt, 12 compounds were detected, among them, the content of trans-RSV and PD was found to be the maximum. The same study also performed the molecular docking of PD and RSV to delineate the pattern of HMG-R inhibitory activity by these compounds. The binding affinities of PD and RSV against the crystal structure of HMG-R were −9.76 kcal/mol and −7.38 kcal/mol, respectively. This study suggested that inhibitory activity on HMG-R by DCM extract of *Gnetum gnemon *L. was due to the existence of significant amount of PD (Hafidz et al., 2017[78]). Another *in silico *study showed that PD inhibited the activity of HMG-R through occupying its active pocket with significant binding energy (∆G: −7.07 kcal/mol) (Artha et al., 2018[21]).

### Naringin

Naringin (4′,5,7-trihydroxyflavanone-7-rhamnoglucoside) (NRN), a natural flavone, is commonly found in the citrus fruits. It has several biological functions including antioxidant, anti-inflammatory, antimutagenic, and hypolipidemic (Jeon et al., 2004[93]; Bacanli et al., 2015[23]; Xulu and Oroma Owira, 2012[214]; Bharti et al., 2014[28]; Cavia-Saiz et al., 2010[39]; Inês Amaro et al., 2009[87]; Thangavel et al., 2012[187]). Animal studies uncovered that NRN has no side effects (Choe et al., 2001[44]). To date, an ample of studies have confirmed the cardioprotective and lipid lowering effects of NRN. Briefly, NRG significantly reduced the level of plasma cholesterol and hepatic TG level via the inhibition of HMG-R activity in rats in a dose-dependent manner and these effects were better than that of reported in lovastatin treated rats (Bok et al., 2000[30]). Another study in HFD-induced hyperlipidemic SD rats showed that administration of NRN for 6-week reduced the plasma cholesterol, TG and hepatic cholesterol content. The same authors also observed the reduced activity of HMG-R in NRN supplemented group (Kim et al., 2006[104]). Another study reported that NRN reduced the LDL level and increased the HDL content in diabetic rats. Additionally, level of hepatic TG and cholesterol were also diminished by the treatment of NRN. The level of HMG-R and ACAT was also reduced with the treatment of NRN (Xulu and Oroma Owira, 2012[214]). Conversely, an *in vivo* study reported that NRN at the dosage of 100 mg increased the hepatic TC and TG content without damaging any hepatocyte in HFD-fed rats (Chanet et al., 2012[41]). Additionally, increasing the dose up to 1.5 g/day reduced the level of plasma TG and cholesterol in lambs (Bodas et al., 2011[29]). The supplementation of NRN to HFD/streptozotocin-induced diabetic rats reduced the serum cholesterol, TG, LDL, and VLDL as well as increased the HDL-C level. Furthermore, NRG treatment also inhibited the activity of HMG-R (Rotimi et al., 2018[168]; Ahmed et al., 2012[9]).

Similarly, several animal studies showed that NRN at different doses reduced the TC, TG, LDL, VLDL and induced the HDL content and also decreased the accumulation of fat droplets in the liver. Furthermore, HMG-R was also decreased in NRN treated type 2 diabetic rats (Alam et al., 2013[11]; Sharma et al., 2011[176]; Pu et al., 2012[158]; Ahmed et al., 2012[9]). The treatment with NRN significantly reduced the oxidative stress level, hepatic TC and TG, plasma insulin, LDL and PCSK-9 in C57BL/6J obese mice. These protective effects of NRN were found to be SREBP-dependent (Sui et al., 2018[184]). Furthermore, a recent molecular docking study revealed that NRG actively binds into the active pocket of HMG-R with significant binding affinity (∆G: −10.75 kcal/mol) (Andrade-Pavón et al., 2021[20]). The treatment with NRN also lowered the inflammation caused by cholesterol in the liver, adipose tissue, and aorta (Assini et al., 2013[22]). The NRN stimulated the expression level of LDL-R mRNA through SREBP-2 and phosphoinositide 3-kinases/extracellular signal-regulated kinase1/2 (PI3K/ERK1/2) pathway (Bawazeer et al., 2017[27]).

### Curcumin

Curcumin (CCM) (1,7-bis(4-hydroxy-3-methoxyphenyl)-1,6-heptadiene-3,5-dione), a natural polyphenolic metabolite majorly identified in turmeric (*Curcuma longa*), has several biological functions such as anti-inflammatory, antioxidant, antimicrobial, anticancer and hypolipidemic (Hewlings and Kalman, 2017[83]; Reddy et al., 2005[165]; Aggarwal and Harikumar, 2009[4]; Lestari and Indrayanto, 2014[118]; Vera-Ramirez et al., 2013[190]; Mahady et al., 2002[134]). Clinically, it has been reported that CCM is safe at high doses in humans. It has shown poor bioavailability, but some formulations have been made to improve its bioavailability like liposomal curcumin, curcumin nanoparticles, and structural analogues of curcumin such as EF24 (Anand et al., 2007[19]; Rachmawati et al., 2015[160]). In an *in vitro* study, CCM induced the expression of LDL-R mRNA expression via the SREBP pathway resulting in increased LDL uptake in HepG2 (Dou et al., 2008[56]). On the contrary, several* in vitro* studies did not support these findings (Peschel et al., 2007[153]; Kang and Chen, 2009[101]). Instead, another study showed that CCM enhanced LDL-R level as well as activity in HepG2 cells, however, LDL-R mRNA expression was not affected. CCM also decreased the PCSK-9 expression level which lead to augmented uptake of LDL in HepG2 cells (Tai et al., 2014[185]). 

In addition, treatment with CCM at the doses of 10 and 20 µM for 24 h reduced HNF-1α which ultimately down-regulated the PCSK-9 mRNA expression as well as protein in hepatic cells. CCM also decreased the level of statin induced PCSK-9 expression (Tai et al., 2014[185]). An *in vivo* study reported that the treatment of CCM (200 mg/kg/d) for 12 weeks significantly reduced both PCSK-9 mRNA expression and protein level and also elevated the level of LDL-R on the surface of hepatocytes in carbon tetrachloride-induced liver cirrhotic SD rats (Cai et al., 2017[33]). Furthermore, an *in vitro *study demonstrated that CCM significantly inhibited the HMG-R activity with an IC_50 _value of 4.3µM (Lin et al., 2015[126]). Another study noted that CCM at the concentration of (2 mg/ml) inhibited the HMG-R activity and the expression of PPARα and LXRα was induced (Rachmawati et al., 2016[161]; Shin et al., 2011[178]). Further, *in vivo* studies demonstrated that CCM ameliorated the hepatic cholesterol and TG level, whereas, level of HDL-C was increased. The FAS and HMG-R activities were also lowered in CCM treated HFD-induced hyperlipidemic rats (Seo et al., 2008[174]; Jang et al., 2008[92]).

Another *in vivo *study showed that the supplementation with CCM reduced the plasma cholesterol, TG and significantly increased the HDL-C level in LDL-R−/− C57BL/6J mice and these effects were similar to the hypocholesterolemic effects of lovastatin. The atherosclerotic lesions also disappeared in CCM treated rats, when compared to untreated rats (Shin et al., 2011[178]). The author also demonstrated that CCM involved in phosphorylation of AMPK and mitigated clozapine-induced overexpression of SREBP. Additionally, CCM also reduced the SREBP-targeted genes responsible for fatty acid synthesis and cholesterol homoeostasis including FAS and HMG-R (Liu et al., 2017[129]).

In the same context, supplementation with tetrahydro-curcumin (80 mg/kg), one of the active metabolites of CCM, reduced the blood glucose level and increased the plasma insulin level in STZ-nicotinamide-induced diabetic rats (Pari and Murugan, 2007[151]). On the other hand, tetrahydro-curcumin also showed the antihyperlipidemic activity leading to reduction in the levels of LDL, VLDL, and TG and significantly reduced the HMG-R activity. Serum HDL level was also restored to normal level after the treatment in diabetic rats (Karthikesan et al., 2010[102]). As previously evident, CCM reduces the liver cholesterol via directly or indirectly binding with HMG-R. In order to validate these findings, a molecular docking study was conducted which revealed that CCM can bind to the active pocket site of HMG-R similar to statins (Islam et al., 2015[89]).

### Lycopene

Lycopene, an intensely colored lipid soluble carotenoid, has great antioxidant activity and is commonly found in tomatoes and other vegetables/fruits (Alvi et al., 2017[15], 2015[17]). It has many biological properties including antioxidant, antidiabetic, anti-inflammatory, anti-atherosclerotic, and plate aggregation inhibition (Costa-Rodrigues et al., 2018[47]; Alvi et al., 2015[17]). Since it is a fat soluble compound, lycopene shows similar absorption as dietary fat (Krinsky and Johnson, 2005[107]). Treatment with lycopene was reported to reduce the level of TC, TG, LDL, and VLDL as well as increased the HDL level in HFD-induced hyperlipidemic rats. (Alvi et al., 2016[18], 2017[16]). It was reported in the same study for the first time that administration of lycopene down-regulated the PCSK-9 and HMG-R mRNA expression in HFD-induced male Sprague-Dawley rats. In addition, the authors also reported the reduced level of inflammatory markers such as TNF-α, IL-1β, IL-6 and C-reactive protein (CRP), which are well reckoned to play a key role in atherosclerotic events (Alvi et al., 2017[16]). The molecular modeling study was also carried out to confirm the PCSK-9 inhibitory activity of lycopene which demonstrated that lycopene restricts the interaction between PCSK-9 and EGF-A complex of LDL-R, whereas, atorvastatin facilitated the PCSK-9-EGF-A complex formation and ultimately the lysosomal degradation of LDL-R (Alvi et al., 2017[16]). Another study showed that lycopene supplementation down-regulates the PCSK-9 expression via suppression of HNF-1α, while the expression of LDL-R was up-regulated in a SREBP-2-dependent mechanism in LPS-challenged rat model infection and inflammation (Alvi et al., 2017[15]). In addition, the expression (mRNA) and activity of hepatic HMG-R was also restricted in lycopene treated rats in rat model of hypercholesterolemia (Alvi et al., 2017[16]) as well as infection/inflammation (Alvi et al., 2017[15]).

Numerous reports have shown the potential HMG-R inhibitory activity of lycopene. A recent study showed that lycopene strongly inhibits the *in vitro* activity of HMG-R with an IC_50_ value of 36 ng/ml, which was significantly lower than that of the reference standard drug, pravastatin (IC_50 _= 42 ng/ml). They further extended their work to determine the mode of inhibition of lycopene against HMG-R activity and showed that lycopene competes with the HMG-CoA, the original substrate for HMG-R, and exhibits competitive mode of inhibition of HMG-R functionality with the K*i* value of 36 ng/ml (Alvi et al., 2016[18]). An *in silico* study also showed that lycopene has higher affinity (ΔG: −5.62 kcal/mol) for the binding site of the enzyme than HMG-CoA (ΔG −5.34 kcal/mol). The authors demonstrated that lycopene used similar binding pocket as the standard drug (pravastatin) against HMG-R. The docked structures of both pravastatin and lycopene with HMG-R were found to be surrounded by Ser865, Lys722, Ala564, Arg568, Val720 and Arg571 amino acid residues, whereas docked structures of HMG-CoA-HMG-R was stabilized by Lys722, Arg568, Gly807 and Glu559. This study showed that lycopene effectively inhibited the HMG-R activity (Alvi et al., 2016[18]).

### Omega 3 fatty acids

The omega-3 (n-3) fatty acids, eicosapentaenoic acid (EPA) and docosahexanoic acid (DHA), are commonly present in seafood (Calder, 2018[34]; Innes and Calder, 2020[88]). EPA and DHA belong to the polyunsaturated fatty acids (PUFAs) family that is also found in several supplements such as fish oil, liver oil of cod, krill oil and some algal oils. They exert number of cardioprotective effects via the regulation of various risk factors involved in the progression of ASCVD like blood lipids, platelet aggregation, endothelial function and inflammation (Innes and Calder, 2020[88]). T cell differentiation is modulated via omega-3 fatty acids which produce several prostaglandins and specialized proresolving lipid mediators that aid in the resolution of tissue injury and inflammation, as well as causing more rapid clearance of LDL particles and slower production of VLDL particles (Mason et al., 2020[137]). These properties are mediated by the suppression of the SREBP-1 regulated pathways such as activiation of HNF-4, Farnesoid X receptor (FXR), liver X receptor (LXR), and PPARs (Pizzini et al., 2017[155]; Scorletti and Byrne, 2018[171]). An *in vivo* study reported that administration of PUFA enriched fish oil reduced the PCSK-9 expression and cocomitantly decreased the plasma LDL-C level by 84 % in SD rats (Yuan et al., 2016[217]). Another *in vivo *study showed that omega-3 fatty acids rich diet reduced the plasma LDL-C, VLDL, and PCSK-9 level in female mice (Sorokin et al., 2016[181]).

Administration of canola oil enriched with DHA reduced the PCSK-9 and TG level in the patients who were suffering from metabolic syndrome (Pu et al., 2016[159]). Clinical intervention of marine PUFAs (38.5 % EPA, 25.9 % DHA) reduced the PCSK-9 level in premenopausal and postmenopausal women by 11.4 % and 9.8 %, respectively (Graversen et al., 2015[72]). These PUFAs are available as drug formulations and dietary supplements. The American Heart Association recommended EPA and DHA for coronary heart disease (CHD) and hypertriglyceridemic patients (Chris Bradberry and Hilleman, 2013[45]). In terms of modulating specific markers of inflammation as well as blood lipids, DHA outperforms EPA (Allaire et al., 2016[12], 2018[13]). For the treatment of serious hypertriglyceridemia, the US FDA has licenced multiple omega-3 formulations that are either concentrated or refined from fish oil. Such combinations which provide EPA and/or DHA in either ethyl ester (EE) need enzyme carboxyl ester lipase digestion. Several techniques were documented to improve the bioavailability of EPA and DHA from EE (Maki and Dicklin, 2019[135]).

A recent clinical study showed that a “pre-digested” OM3-sn-1(3)-monoacylglycerol lipid structure (OM3-MAG) has markedly augmented absorption at high doses (2.9 g/day) when compared with commonly used OM3-ethyl ester (3.1 g/day) form used for the treatment of high TG level. Therefore, the use of pre-digested OM3-MAG is recommended as a more effective therapy in extreme cardiovascular situations, where high omega-3 doses are necessary and a low-fat diet is recommended (Cuenoud et al., 2020[50]). Numerous studies (*in vivo *and *in vitro*) showed that PUFAs are effective inhibitors of HMG-R activity and are useful in the treatment of hyperlipidemia and hypercholesterolemia (El-Sohemy and Archer, 1997[63], 1999[62]; Duncan et al., 2005[59]; Das, 2001[54]; Nakamura et al., 1998[147]). In an *in vitro *study, the researchers showed that PUFAs also diminished the expression of SREBP-2 and down-regulated the expression of HMG-R mRNA in H4IIEC3 rat hepatoma cells (Le Jossic-Corcos et al., 2005[116]). Additionally, in the same study, EPA and DHA decreased the expression of SREBP-2 in human HepG2 hepatoblastoma cell. These findings suggested that PUFA can suppress the hepatic cholesterol synthesis through inhibition of HMG-R and the impairment of the SREBP-2 pathway (Le Jossic-Corcos et al., 2005[116]).

### Emodin

Emodin (6-methyl-1,3,8-trihydroxyanthraquinone), an anthraquinone extracted from *Rheum palmatum* L. (Polygonaceae) and several Chinese herbs (Shang and Yuan, 2002[175]). A very recent *in vivo *study showed that administration of emodin for 10 days reduced the TC, TG, LDL-C levels and increased the HDL-C level in a dose-dependent manner in HFC-fed zebra fish larvae. Additionally, to detect the underlying mechanism of hypolipidemic effect of emodin, relevant gene expression in lipid metabolism was also studied. After 10 days of emodin administration, the expression of AMPKα, LDL-R, ABCA1 and ABCG1 were remarkably stimulated and SREBP-2, PCSK-9 and HMG-R were suppressed. The authors also reported that the liver function was improved as the ALT and AST levels were decreased following the emodin treatment (He et al., 2021[82]). Another *in vivo* study reported that emodin (10 mg/kg) significantly reduced the TC, TG, and LDL levels where LDL-C reduction via emodin was near to the simvastatin in HFD-induced hypercholesterolemic rats. Moreover, the LDL-R expression was significantly induced by emodin administration (Wu et al., 2021[207]). Emodin (40 mg/kg/day and 80 mg/kg/day) reduced the lipid level in HFD-induced C57BL/6 mice (Li et al., 2016[120]). 

A very recent study showed that aloe, which also contains emodin, reduced the TC and LDL-C levels in diet-induced hypercholesterolemic rats (100 mg/kg per day of aloe). Infact, aloe also attenuates the fat content of liver. On the other hand, an *in vitro* study on HepG2 cells suggested that aloe had false impact on SREBP and HNF1α, which ultimately led to the down-regulation of PCSK-9 as well as increased availability of LDL-R to facilitate the LDL uptake (Su et al., 2020[183]). An *in vitro* study showed that emodin reduced the TC and triglycerides in steatosis hepatocyte L-02 cells via down-regulation of HMG-R and up-regulation of CYP7A contents after 48 h exposure (Wang et al., 2014[197]). A cell-based study reported that emodin treatment inhibited the SREBPs transactivity in huh7 cell line. Further, emodin treatment significantly reduced SREBP-1 and SREBP-2 mRNA expressions in the liver and adipose tissue (Li et al., 2016[120]). Another *in vitro *study of *Cassia mimosoides *Linn extract that contains emodin showed that emodin (1 and 5 μg/mL) significantly decreased HMG-R activity, when compared to pravastatin (Shen et al., 2016[177]). An *in vivo* study also revealed that emodin significantly reduced the expression of HMG-R in HFD-induced hyperlipidemic male C57BL/6 mice (Li et al., 2016[120]).

### Eugenol

Eugenol (4-allyl-2-methoxyphenol), a natural phenolic compound commonly present in clove (*Syzygium aromaticum* L.), ) is a medicinal plant in the family *Myrtaceae* known for its traditional use as a spice (Harb et al., 2019[80]; Adorni et al., 2020[3]). The daily consumption of eugenol up to 2.5 mg/kg body weight has been recommended by the WHO as safe for human use. Several *in vivo* studies demonstrated that eugenol decreases the serum and hepatic cholesterol level and also prevents lipogenesis, thus suggesting a protective effect against atherosclerosis and fatty liver disease (Jo et al., 2014[100]; Elbahy et al., 2015[60]). Eugenol significantly reduced the TC, TG, LDL level and improved the liver function via decreasing hepatic markers like ALT and AST. In an *in vitro* study eugenol reduced the PCSK-9 and LOX-1 expression in Jurkat cells. Computational molecular modeling study showed that LOX-1 stably interacted to PCSK-9 with Ile149, Leu157 and Phe158 amino acid residues. In case of eugenol, amino acid residues Arg495, Asp570, and Leu571 involved in the hydrophobic interactions with PCSK-9 (Zia et al., 2020[221]). Eugenol did not inhibit the hepatic HMG-R activity but down-regulated the transient receptor potential vanilloid (TRPV1) channels in the liver (Harb et al., 2019[80]). However, an *in silico* study showed that eugenol binds with the active pocket of HMG-R with strong hydrogen bonding interaction and showed a great binding score of -79.4759 (Muthusamy et al., 2017[141]).

### Ginkgolide B

Ginkgolide B (GB), a natural terpene lactone, has pharmacological potential benefits in the cardiovascular system (Wang et al., 2019[194]). An *in vivo* study showed that TC, TG, and LDL-C levels were decreased and HDL-C levels were increased in C57BL/6 mice administered GB. The expression of SREBP-2, acetyl-CoA carboxylase (ACC) and FAS was also remarkably inhibited (Luo et al., 2017[133]). In ox-LDL-treated human umbilical vein endothelial cells (HUVECs) and RAW246.7 murine macrophages, GB inhibits lectin-type oxidized LDL receptor 1 (LOX1) expression, resulting in lower cholesterol accumulation in HUVECs and RAW264.7 (Feng et al., 2018[65]; Wang et al., 2019[194]). A very recent *in vitro *study investigated the effect of GB on incubated ox-LDL-exposed HUVECs. Ox-LDL responsible for the up-regulation of expression of PCSK-9 in HUVECs. After treatment of GB, PCSK9 suppressed at both mRNA and protein level, SREBP-2 expression and inflammatory cascades were also inhibited. In the same study the authors also validated GB-mediated inhibition of PCSK-9 activity by *in silico* methods, which revealed that it interferes the PSCK-9 interaction with EGF-A of LDL-R (Wang et al., 2019[194]). 

A detailed searching of articles revealed that presently there is no data regarding the impact of GB on HMG-R activity. However, *Ginkgo biloba* extract (GBE) is known to modulate the cholesterol metabolism via decreasing the blood cholesterol level in cholesterol-enriched diet fed rats and rabbits (Yao et al., 2004[215]; Igarashi and Ohmuma, 1995[86]; Lin et al., 2002[127]). In cell line experiment GBE also modulates cholesterol levels in HepG2 and SK-HEP-1 cells. The treatment with GBE reduced the TC content in a dose-dependent manner in both HepG2 and SK-HEP-1 cells. GBE also reduced the activity of HMG-R by 6.2 %, 16 % and 71.6 % at concentrations of 32.26, 322.6, and 3226 μg/mL, respectively. The bioactive component of GBE are ginkgolides A and B that attributed to its cholesterol-lowering effect via inhibition of HMG-R activity (Xie et al., 2009[212]).

### Luteolin 

Luteolin (3,4,5,7-tetrahydroxyflavone) is one of the most common flavones, found in a number of vegetables, fruits, and herbs, including carrots, cabbage, artichokes, tea, celery, and apple (Weng and Yen, 2012[202]; Wang et al., 2009[195]). Luteolin is readily absorbed by the intestinal mucosa. After the oral administration of 14.3 mg/kg luteolin, the maximum plasma concentration (C_max_) was reported to 1.97 ± 0.15 mg/ml, which was achieved in 1.02 ± 0.22 h with the half-life (t_1/2_) 4.94 ± 1.2 h. It was converted to free luteolin, glucuronide and sulfate-conjugates of luteolin and of O-methyl luteolin (diosmetin or chrysoeriol) after metabolism in rats. Free luteolin has also been reported in human plasma (Lopez-Lazaro, 2008[130]). An *in vitro* study revealed that expression of SREBP-2 in WRL and HepG2 cells was suppressed via the luteolin treatment. It is also known to block the nuclear translocation of SREBP-2 which was essential for nuclear translocation and consequent modulation of genes necessary for cholesterol homeostasis. Luteolin partially blocked the activation of SREBP-2 through increased AMP kinase (AMPK) activation and also suppressed the SREBP-2 expression.

It was further validated by reporter gene assays that the transcription of SREBP-2 was weakened in response to luteolin. The diminished expression and protein processing of SREBP-2 is demonstrated in reduced nuclear translocation and results in decreased transcription of HMG-R after luteolin treatment (Wong et al., 2015[206]). HMG-R activity was clearly lower in luteolin (125 μg/mL) than Pravastatin (125 μg/mL) (Shen et al., 2016[177]). An *in vivo *study showed that luteolin decreased the atherosclerosis in LDL-R deficient C57BL/6 mice suggesting that luteolin stimulates the LDL-R gene expression (Jiang Li et al., 2018[97]; Ochiai et al., 2016[150]). Luteolin-7-glucoside, one of the luteolin glycosidic form, also reduced the expression of HMG-R but did not affect the SREBP-2 and LDL-R mRNA expression (Sá et al., 2015[169]). Moreover, an *in silico* study showed that luteolin strongly binds to the HMG-R and also the K*i* showed that luteolin can be used as potent HMG-R inhibitor and may act as a novel antihyperlipidemic agent (Nematollahi et al., 2012[148]). Another *in silico* study also showed that luteolin strongly binds with HMG-R with a very high interaction energy (∆G: -61.6 kcal/mol), while such strong interaction was facilitated by Lys549, Gly577, Ser580, Arg582, and Arg540 residues of HMG-R (Radhakrishnan et al., 2018[162]).

## Conclusion

**Figure 3:**
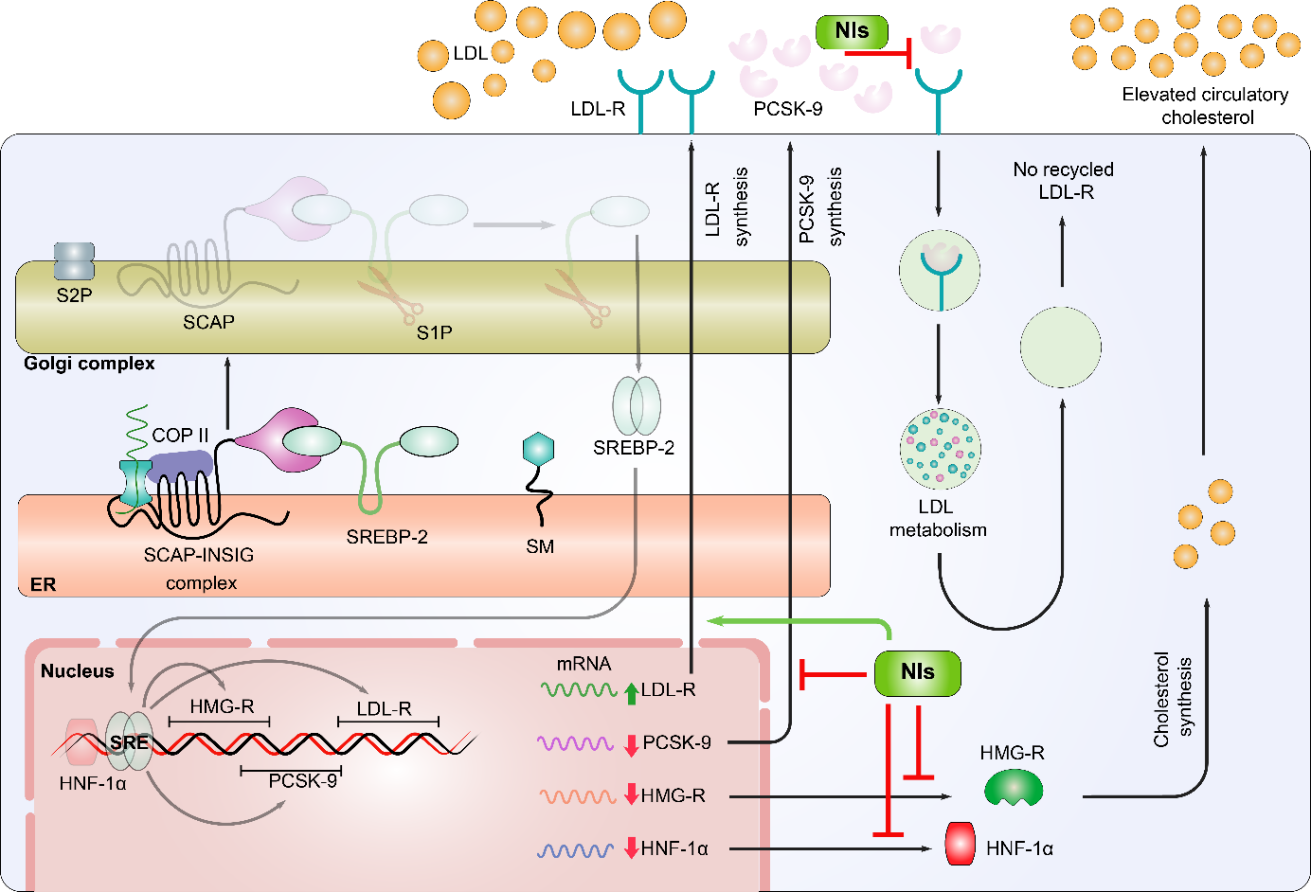
Potential mechanisms of inhibition of PCSK-9 and HMG-R via natural inhibitors (NIs). Natural compounds adopt distinct mechanisms to maintain the cholesterol biosynthesis and to prevent the atherosclerosis. These mechanisms are: Natural compounds are involved in the up-regulation of LDL-R mRNA expression and down-regulation of PCSK-9, HMG-R, and HNF-1α expression. Level of LDL-R protein is enhanced on the surface of hepatocytes and hence the recycling of LDL-R is also stimulated. The implication of NIs enhanced the LDL-C clearance through LDL-R and also restricted the interaction between PCSK-9 and LDL-R.

Dysregulation of cholesterol and lipoproteins, particularly elevated LDL-C, have been linked with greater risk of ASCVD. Considering the regulatory role of HMG-R and PCSK-9 in cholesterol homeostasis, both of these biomarkers have been established as the preferred targets in ASCVD risk management. However, the undesirable adverse outcomes of classical HMG-R and PCSK-9 inhibitors have demanded the discovery of alternative potential dual inhibitors of HMG-R and PCSK-9 from natural sources. Therefore, the initial part of the current report explains about the strict regulation of cholesterol metabolism in response to the availability of extracellular cholesterol thresholds, while the remaining one compiles the pharmacological effects of natural secondary metabolites with special emphasis on their dual HMG-R and PCSK-9 inhibitory action (Figure 3(Fig. 3)). In conclusion, various natural products exhibit atheroprotective effects via targeting HMG-R and PCSK-9 activities and lipoprotein metabolism, however, further clinical assessments are still warranted prior their approval for ASCVD risk management in hypercholesterolemic patients. 

## Declaration

### Acknowledgment

Authors are thankful to the Honorable Chancellor for providing state-of-the art research facility at Integral University. Authors are also indebted to the Department of Health Research (DHR), Ministry of Health & Family Welfare (MoHFW), GOI, for providing grant (File No. 12014/13/2019-HR). This manuscript has the institutional manuscript communication No. IU/R&D/2021-MCN0001296.

### Conflict of interest

Authors declare that they have no conflict of interest.

### Author contributions

Conceptualization: M.S.K.; Literature Review & preparation of manuscript draft: M.W.; Editing of the draft manuscript: M.S.K. & S.S.A.; Illustrations: M.W. & S.S.A.; Final revision & approval of the manuscript: M.S.K.
